# Adaptation and implementation of the WHO Safe Childbirth Checklist around the world

**DOI:** 10.1186/s43058-021-00176-z

**Published:** 2021-07-08

**Authors:** Rose L. Molina, Anne-Caroline Benski, Lauren Bobanski, Danielle E. Tuller, Katherine E. A. Semrau

**Affiliations:** 1 Ariadne Labs, Harvard T.H. Chan School of Public Health/Brigham and Women’s Hospital, Boston, MA USA; 2 Department of Obstetrics and Gynecology, Beth Israel Deaconess Medical Center, 330 Brookline Ave, Kirstein 3rd floor, Boston, MA 02215 USA; 3 Department of Medicine, Harvard Medical School, Boston, MA USA; 4 Department of Women, Child and Adolescent, University Hospital of Geneva, Geneva, Switzerland; 5 Harvard T.H. Chan School of Public Health, Boston, MA USA; 6 Division of Global Health Equity, Brigham and Women’s Hospital, Boston, MA USA

**Keywords:** Safe Childbirth Checklist, Maternal health, Quality of care, Adaptation, Implementation

## Abstract

**Background:**

The World Health Organization (WHO) published the WHO Safe Childbirth Checklist in 2015, which included the key evidence-based practices to prevent the major causes of maternal and neonatal morbidity and mortality during childbirth. We assessed the current use of the WHO Safe Childbirth Checklist (SCC) and adaptations regarding the SCC tool and implementation strategies in different contexts from Africa, Southeast Asia, Europe, and North America.

**Methods:**

This explanatory, sequential mixed methods study—including surveys followed by interviews—of global SCC implementers focused on adaptation and implementation strategies, data collection, and desired improvements to support ongoing SCC use. We analyzed the survey results using descriptive statistics. In a subset of respondents, follow-up virtual semi-structured interviews explored how they adapted, implemented, and evaluated the SCC in their context. We used rapid inductive and deductive thematic analysis for the interviews.

**Results:**

Of the 483 total potential participants, 65 (13.5%) responded to the survey; 55 completed the survey (11.4%). We analyzed completed responses from those who identified as having SCC implementation experience (*n* = 29, 52.7%). Twelve interviews were conducted and analyzed. Ninety percent of respondents indicated that they adapted the SCC tool, including adding clinical and operational items. Adaptations to structure included translation into local language, incorporation into a mobile app, and integration into medical records. Respondents reported variation in implementation strategies and data collection. The most common implementation strategies were meeting with stakeholders to secure buy-in, incorporating technical training, and providing supportive supervision or coaching around SCC use. Desired improvements included clarifying the purpose of the SCC, adding guidance on relevant clinical topics, refining items addressing behaviors with low adherence, and integrating contextual factors into decision-making. To improve implementation, participants desired political support to embed SCC into existing policies and ongoing clinical training and coaching.

**Conclusion:**

Additional adaptation and implementation guidance for the SCC would be helpful for stakeholders to sustain effective implementation.

**Supplementary Information:**

The online version contains supplementary material available at 10.1186/s43058-021-00176-z.

Contributions to the literature

- This study provides insight into the variation in adaptations and implementation strategies among users of the World Health Organization’s Safe Childbirth Checklist since its dissemination in 2015.
- Adaptations to the Checklist content were common and incorporated local guidelines and end-user feedback. Implementation strategies also varied but were constrained by funding and infrastructure limitations.
- Many implementers did not have the funding or capacity to perform a rigorous impact evaluation of the Checklist implementation, and the implementation duration varied across sites.
- Additional guidance on how to adapt and implement the Checklist in a variety of settings with different contexts is needed.

## Background

To reduce maternal and perinatal morbidity and mortality, the World Health Organization (WHO) led the development of the WHO Safe Childbirth Checklist (SCC), a patient safety tool that includes the essential practices that should be performed during facility-based childbirth [1]. The WHO Patient Safety Programme; WHO Department of Maternal, Newborn, Child, and Adolescent Health; WHO Department of Reproductive Health and Research; and Harvard T.H. Chan School of Public Health convened more than 50 international experts in maternal and newborn health and developed the SCC through a systematic step-wise process in 2008–2009. The WHO SCC was designed to address the leading causes of maternal and perinatal morbidity and mortality; its development included review of guidelines and evidence, iterative refinement through consultation with a broad stakeholder network, and field testing in 9 high-priority settings [2].

The WHO made the SCC publicly available in 2015. Researchers have used the SCC in a variety of contexts and have reported favorable impacts on quality of care and outcomes [3, 4]. The BetterBirth Trial, the largest study of SCC implementation, took place in 120 primary facilities in Uttar Pradesh, India, between 2014 and 2017 and enrolled over 157,000 woman-newborn pairs [5]. The study demonstrated that implementation of the SCC with an 8-month peer-coaching program and continuous data feedback led to increased adherence to essential birth practices, but did not reduce maternal or perinatal severe morbidity or mortality [5]. A post hoc analysis from the BetterBirth trial demonstrated that provider adherence to a high number of SCC practices was associated with reduced perinatal mortality [6]. A recent cluster-randomized controlled trial in Uganda and Kenya demonstrated reductions in fresh stillbirth and neonatal mortality when a modified SCC was implemented as part of a package including data strengthening, team training, and quality improvement collaboratives [4]. A systematic review of the SCC’s impact on essential birth practices and outcomes showed that there is moderate quality evidence that utilization of the SCC is effective in reducing stillbirth and improving some essential birth practices, such as management of pre-eclampsia and maternal infection [7].

In order to assess feasibility and acceptability of SCC implementation, the WHO created the WHO SCC Collaborative, which included implementation teams from 39 sites across 19 countries, to explore barriers and facilitators of implementing the SCC in a variety of contexts between 2012 and 2015 [8]. Key recommendations emerging from the WHO SCC Collaborative included the importance of engaging key stakeholders, assessing competency of end-users and providing technical skills training when necessary, and facilitating local adaptation of the SCC with ongoing supervision and support [8]. However, there lacks consolidation of the different strategies for SCC adaptation and implementation since the formation of the WHO SCC Collaborative. The aim of our study was to assess the current use of the SCC, the range of adaptations regarding the SCC tool and implementation strategies in different contexts, and the facilitators and barriers to ongoing use.

## Methods

This was an explanatory, sequential mixed methods study of global SCC implementers, which included a survey and follow-up interviews. We used a mixed methods design to merge data from a diverse group of implementers and explore tool adaptations and implementation strategies in greater depth with a subset of respondents. Quantitative data from survey responses were used to guide interview discussions about adaptation and implementation experiences, including facilitators and barriers to implementation. The Harvard T.H. Chan School of Public Health Institutional Review Board determined this study qualified as quality improvement and was not considered human subjects research. Ariadne Labs, a joint center for health system innovation between Harvard T.H. Chan School of Public Health and Brigham and Women’s Hospital, provided internal funding for this study.

### Survey

We created a 20-min survey about the respondent’s experience with the SCC, including adaptations and implementation strategies, data collection about SCC use, and thoughts about a community of practice and other resources needed to support ongoing SCC implementation. The items about implementation strategies were based on the WHO SCC Implementation Guide [9] with free-text options for the respondents to include additional information if the options did not match their experience. There were up to 30 items included based on branching logic, 7 of which were open-ended. Between February and April 2020, we sent the survey via Qualtrics email link to 451 maternal-child health program leads and implementers who had contact with Ariadne Labs. We sent the survey to 32 additional implementers identified through snowball sampling for a total of 483 potential participants. Inclusion criteria were any person who interacted with Ariadne Labs regarding the SCC (e.g., colleagues, people who left business cards, or signed up for information at conferences) and who had a valid email address in Ariadne Labs records. We relied on the Ariadne Labs professional network for survey dissemination because of the organization’s leadership in co-designing, evaluating, and spreading the SCC. Potential respondents received a secure email link and three weekly reminders if they had not completed the survey.

We analyzed the survey results using descriptive statistics in Microsoft Excel and reported frequencies and percentages. One organization developed a mobile app for the SCC and was not involved in any on-site implementation, so it was excluded from some analyses specific to implementers. The lead investigator conducted thematic analysis of the 7 open-ended responses with formal coding to evaluate each response through an inductive approach in Microsoft Excel. The themes included SCC adaptations and decision making; initial and ongoing SCC training; and data collection, analysis, and use of findings. Any uncertainty in assigning codes was settled during discussion with the principal investigator and other study staff.

### Semi-structured interviews

The quantitative results from the survey were further explored with a subset of respondents who identified as implementers in follow-up qualitative semi-structured interviews about how they adapted, implemented, and evaluated the SCC. We interviewed all respondents who consented to participate. We explored specific implementation strategies around the SCC consistent with those compiled in the implementation science literature [10]. Additional interview topics included how to build a community of practice to disseminate implementation experiences. Virtual interviews through Zoom lasted 60–90 min and were recorded, summarized, and analyzed using a rapid qualitative approach [11]. The lead author, a female obstetrician-gynecologist with training in public health and qualitative research, led the interviews and disclosed this background to participants. A study staff member took comprehensive notes during the interviews, and the interviewer and note-taker reviewed the notes immediately following the interview to ensure accuracy. Interview segments that required additional review were noted and the recordings were reviewed to ensure accuracy.

The lead investigator used a combination of inductive and deductive approaches to create the codebook using Dedoose software (Version 8.0.35, Los Angeles, CA: SocioCultural Research Consultants, LLC www.dedoose.com). The lead investigator grouped codes into themes based on the study objectives and identified emerging thematic linkages within these content areas. We mapped the implementation challenges that emerged from the interviews to the Consolidated Framework in Implementation Research (CFIR) because of its comprehensive inclusion of implementation factors for a multi-level intervention [12]. Given the exploratory nature of identifying implementation challenges, the CFIR provides a complete set of domains and factors to apply standard language and map the themes that emerged through the inductive approach. We used a deductive approach based on a priori themes around SCC adaptation and evaluation and desired functionality of a community of practice. To integrate the data, the study team reviewed and discussed the quantitative survey findings and the evolving codebook from the qualitative data using inductive and deductive approaches; any discrepancies in the codebook were resolved through consensus.

## Results

**Fig. 1:**
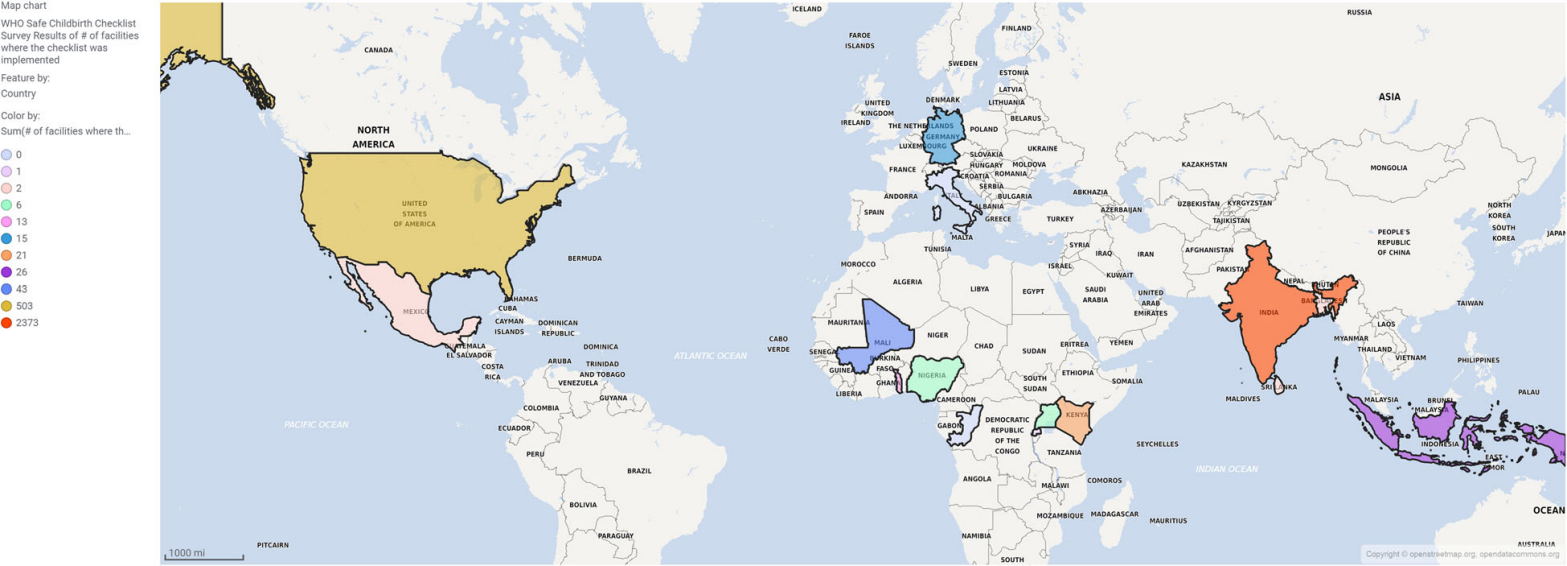
Map of Countries where Survey Respondents Implemented the WHO Safe Childbirth Checklist

Of the 483 total potential participants (451 from original sample and 32 from snowball sampling), 65 people responded to the survey (13.5%) and 55 of those completed the survey (11.4%). In the analysis, we included completed responses from those who reported having implemented the SCC or who planned on implementing it in the upcoming 12 months (*n* = 29). Respondents who implemented the SCC were from 15 countries (Fig. 1). The 26 respondents who completed the survey and did not report implementation experience mostly came from the non-profit sector and academic institutions, similar to the distribution of implementers who were included in the analysis. The majority of respondents were from non-profit organizations or academic institutions, followed by the private and public sectors (Table 1). Ten organizations (35.7%) implemented the SCC in 1–10 facilities, and 9 of those organizations (32.1%) are currently using the SCC (Supplemental Figure 1). There were only 3 organizations (10.7%) that were actively using the SCC when implementation was scaled to 11–1000 facilities (Supplemental Figure 1).

**Table 1:** Survey participant and SCC implementation characteristics

	***N*** = 29, n (%)
**Participant’s organization type**^a^	
Non-profit organization	15
Academic institution	12
Private sector	3
Government or public sector	2
Other	1
**Type of facility where SCC implemented**	
Primary health facilities only	4 (13.8)
Secondary level facilities only	3 (10.3)
Tertiary level facilities only	4 (13.8)
Primary health facilities and secondary level facilities	9 (31.0)
Primary and tertiary level facilities	1 (3.5)
Secondary and tertiary level facilities	2 (6.9)
Primary, secondary and tertiary level facilities	5 (17.2)
Other: App for medical education	1 (3.5)
**Year SCC was first implemented**	
2010	1 (3.4)
2012	4 (13.8)
2014	3 (10.3)
2015	6 (20.7)
2016	9 (31.0)
2017	2 (6.9)
Unknown	4 (13.8)

^a^Not mutually exclusive categories

### Adaptations to the Checklist content and structure

The majority of survey respondents (*n* = 26/29, 90%) and interview participants indicated that they adapted the SCC tool. Examples of adaptations to SCC content included adding clinical items (harmonization with national or regional guidelines, gestational age dating, triage evaluation, blood product availability, management of preterm labor and birth, respectful care practices, appropriate use of corticosteroids and tocolytics, need for anti-malarial medication, newborn anthropometrics, kangaroo mother care, postpartum contraception, and referral assessment) and operational items (confirmation practice was done, names and signatures of people involved in care, supply inventory, discharge summary, and transportation arrangement). Adaptations to the SCC structure included changes to the form (translation into local language, formatting on single page, inclusion of WHO Safe Surgery Checklist, incorporation into a mobile app) and function (integration into the medical record, different versions for physicians, midwives, and nurses) (Supplemental Table 1). The process of adapting the SCC often included consultation with key stakeholders, including technical advisors, government leaders, facility leaders, and clinicians.

### Adaptations to implementation

Survey respondents reported variation in implementation activities summarized in the WHO SCC Implementation Guide (Table 2). In the Engage phase, the majority of participants reported adapting the SCC to match local guidelines and protocols (79.3%) and met with stakeholders to obtain buy-in for the SCC (75.9%). In the Launch phase, the majority of participants incorporated technical training to address gaps in competency (79.3%) and held an official launch event for the SCC (75.9%). In the Support phase, the majority of participants reported observation and coaching strategies to motivate behavior change (86.2%) and shared information regularly with frontline clinical staff (82.8%). The most common barriers in implementing the SCC were staff skepticism about the importance of the SCC (64.3%), SCC not integrated into routine workflow (50.0%), and SCC perceived as burdensome (50.0%) (Table 3). The most salient facilitators of SCC implementation were leadership commitment (57.1%), capacity for quality improvement (42.9%), and organizational culture of accountability, staff appreciation, and openness to change (42.9%) (Table 3).

**Table 2:** Implementation activities from WHO SCC Implementation Guide

Implementation activities from WHO Implementation Guide	***N*** = 29, n (%)
**Engage**	
Adapt the Checklist to fit local guidelines and protocols	23 (79.3)
Meet with stakeholders to obtain buy-in for Checklist implementation	22 (75.9)
Review current resources and practices to determine what is needed for the Checklist to be successful	20 (69.0)
Establish a team to take ownership of the Checklist	6 (20.7)
Supportive supervision and advocacy	2 (6.9)
**Launch**	
Incorporate technical training to address gaps in knowledge, practice, or attitudes	23 (79.3)
Official launch of the Checklist through a special event or training	22 (75.9)
Use SCC framework during antenatal care visit	1 (3.4)
**Support**	
Observing Checklist use and using coaching skills to give respectful and constructive feedback to encourage change and motivate adherence	25 (86.2)
Sharing information regularly to encourage improvement	24 (82.8)
Documenting successes and challenges by gathering information on use of the Checklist, essential birth practice behaviors and supply availability	22 (75.9)
Discussing Checklist use and showcasing people in the facility using the Checklist	21 (72.4)
Assessing availability of essential resources	1 (3.4)

**Table 3:** Barriers and facilitators of SCC implementation

	***N*** = 28 n (%)
**Barriers**	
Skepticism about importance or value among staff	18 (64.3)
Checklist use not integrated into routine workflow	14 (50.0)
Checklist perceived as burdensome	14 (50.0)
Lack of enabling environment (lack of resources, medications, equipment)	13 (46.4)
Lack of training, coaching, or supportive supervision	12 (42.9)
Lack of leadership support for Checklist	7 (25.0)
Lack of staff	1 (0.04)
**Facilitators**	
Leadership commitment	16 (57.1)
Capacity for quality improvement (identifying a local champion, ability to collect and share data)	12 (42.9)
Organizational culture including accountability, staff appreciation, openness to change	12 (42.9)
Adequate skills and training of staff	9 (32.1)
Sufficient staffing	8 (28.6)
Supply availability	8 (28.6)
Facility commitment to respectful patient care	7 (25.0)
Physical condition of facility	2 (7.1)
Effective communication within a facility and across prenatal/ postnatal services	2 (7.1)
Patient and community empowerment	1 (3.6)
Community practices, beliefs and knowledge	1 (3.6)

We asked implementers about how, if at all, data has been used to demonstrate the impact of SCC implementation and inform ongoing SCC use. The majority of implementers reported collecting data around SCC use (72%). The types of data collected varied across sites. Types of data collected included routine clinical indicators from medical records (e.g., maternal/perinatal morbidity and mortality, complications, and mode of delivery), adherence to practices based on SCC audits or direct observation, competency assessments, user perceptions of acceptability and satisfaction with SCC, and inventory of facility infrastructure (e.g., beds, personnel, supplies, medications).

### Implementation challenges and successful strategies

We interviewed 12 implementers from 10 countries to explore adaptations of the SCC, implementation strategies, and reflections about implementation challenges and successful strategies. Participants had experience from management, implementation, clinical care, and/or research. Most participants (*n* = 9) reported that improving safety and quality of childbirth care was the driving reason for implementing the SCC. Other reasons included improving patient satisfaction with care, obtaining hospital accreditation, and complying with government mandates.

Interview participants described various implementation strategies in both the initial and continuing support phases (Supplemental Table 2). Initial implementation included preparation (engaging facility leadership and ensuring required resources were available prior to implementation) and training around SCC use, which was sometimes paired with additional technical training such as emergency obstetric care, simulation training, and newborn resuscitation. Continuing support approaches included regular on-site mentorship or coaching, group text messaging for troubleshooting, refresher training, and regular safety or quality review meetings.

Implementation challenges and successful strategies cited during interviews were mapped to the 5 domains of the CFIR (intervention characteristics, inner setting, outer setting, characteristics of individuals, and implementation process) and their respective constructs (Table 4) [12]. Responses spanned all challenge constructs, but the most salient challenges centered on lack of an enabling environment to support ongoing SCC use. One participant explained:

As soon as patients come, I know the things that I need. But [the Checklist] hasn’t helped me in getting them. It hasn’t helped me in getting improved funding. I think the bottom line is funding. In the initial time, the bottom line wasn’t funding. The bottom line was problems with the quality of care, things that you forget to do for the patients, so in that regard the Checklist is still very valuable. But after awhile, after continuous use, after you have reached the number of people providing care and they have become champions of the checklist, it’s not as useful for them because it doesn’t make the drugs to be available. It doesn’t make water supply to be there. It doesn’t make electricity. It doesn't increase the amount of theater space you have, so it comes back to that cycle of frustration again. [Participant from an academic setting]

**Table 4:** SCC implementation challenges and successful strategies with associated Consolidated Framework for Implementation Research challenge construct and domain

**Intervention challenges**	**CFIR challenge construct**
Dependence on external funding for sustainability (NGOs)	● Intervention source ● Cost
Funding for ongoing mentorship/coaching	● Intervention source ● Cost
Effective coordination and structure of mentorship/coaching	● Design quality and packaging
SCC not designed for teams	● Adaptability
SCC not integrated into medical record	● Adaptability
SCC perceived to be tool for LMICs only	● Relative advantage
Cost of printing SCC and missing SCC	● Complexity ● Cost
Inability to adapt SCC due to government mandate	● Adaptability
**Outer setting challenges**	**CFIR challenge construct**
Government does not enforce or support SCC	● External policy and incentives
Patient care seeking behavior and preferences	● Patient needs and resources
Lack of timely referral system	● Patient needs and resources ● Cosmopolitanism
Incentives required for motivation	● Peer pressure ● External policy and incentives
Tension between data collection for research and sustainable implementation	● Cosmopolitanism ● Patient needs and resources ● External policy and incentives
**Inner setting challenges**	**CFIR challenge construct**
Tension and interpersonal dynamics between different cadres	● Networks and communications ● Culture
Lack of leadership support for SCC	● Readiness for implementation
Staff turnover	● Readiness for implementation
Lack of required infrastructure (personnel, supplies, space)	● Structural characteristics ● Readiness for implementation
Quality of care not perceived as a priority	● Implementation climate ● Readiness for implementation
**Characteristics of individuals challenges**	**CFIR challenge construct**
Lack of motivation and perceived burden of SCC	● Knowledge and beliefs about the intervention ● Other personal attributes
Gaps in technical knowledge/skills in labor management	● Self-efficacy ● Other personal attributes
Resistance to behavior change	● Individual stage of change
**Process challenges**	**CFIR challenge construct**
No clear process for evaluation or audit of individuals	● Reflecting and evaluating
Difficult to use SCC in emergency situations	● Executing
SCC not integrated into routine workflow	● Planning ● Executing
No clear mechanism for identifying once practices have become habit without SCC	● Reflecting and evaluating
**Successful strategies**	**CFIR domain**
● Incorporate accountability into SCC documentation and implementation	Intervention
● Government policy or mandate for SCC	Outer setting
● Include birth companions in care delivery ● Link SCC implementation to other structural changes at facility ● Strengthen health facility infrastructure to accomplish SCC behaviors	Inner setting
● Develop motivational strategy around SCC ● Long-term external coach/supervisor who has support from leadership and frontline clinicians ● Include ongoing technical training to address gaps in knowledge/skills	Characteristics of individuals
● Embed oversight of SCC to ensure it is used with high quality ● Create supporting documentation to facilitate SCC use (discharge warning signs) ● Engage leaders at facility and district levels before implementation ● Learn from a model facility where SCC was implemented successfully (either locally or internationally) ● Mentoring/coaching system to support ongoing SCC use ● Incorporate feedback continuously ● Integrate SCC into workflow ● Integrate SCC into medical record	Process

Successful strategies spanned all implementation domains, and the most frequent ones were reported around the implementation process. Examples include creating supporting documentation to facilitate SCC use (e.g., discharge warning signs), mentoring/coaching system to support ongoing use, integrating SCC into workflow, and incorporating feedback continuously.

### Desired changes to the Checklist

We asked interview participants to describe any desired improvements they would like to see in the SCC content, structure, and implementation based on their experiences. The most salient improvements to SCC content were clarifying the purpose of the SCC, adding guidance on relevant clinical topics, refining items addressing behaviors with low adherence, and integrating contextual factors into decision-making (Table 5). With regard to the SCC structure, participants desired ways to maximize ease of use and to emphasize quality of care. With regard to SCC implementation, participants desired guidance to improve initial and continuous implementation, including the appropriate political support to embed SCC into existing policies and ongoing clinical training and coaching.

**Table 5:** Desired improvements to SCC structure, content, and implementation

**Desired improvements to SCC content**	**Examples**
Clarify purpose of SCC for users	- Decision support tool - Data collection tool - Accountability tool - Quality improvement tool
Add relevant clinical topics and patient information^a^	- Management of preterm birth - Newborn care - Patient demographics
Iterate on items for essential practices with low adherence or inappropriate practices	- Vital signs measurement - Hand hygiene - Augmentation of labor without medical indication - Inappropriate fundal pressure
Integrate contextual factors into SCC decision-making	- Patient/family preferences regarding referral - Managing multiple concurrent deliveries - Incorporate feedback from frontline clinicians
Update SCC items to reflect current WHO initiatives	- Sustainable Development Goals - Universal Health Coverage - Quality of Care Network
**Desired Improvements to SCC Structure**	**Examples**
Ease of use	Digital version of SCC Integration into medical record Translation into local languages Redesigned format (not a checklist)
Emphasis on quality of care	Separation of SCC from medical record to emphasize ongoing supportive processes to enable behavior change
**Desired improvements to SCC Implementation**	**Examples**
Initial implementation support	Political support to embed SCC into existing policies Guidance for considering contextual factors in decision-making Guidance on how to select ideal SCC pilot sites
Continuous implementation support	Additional clinical training in management of complications Patient-centered care and experience of care Collaboration between public and private sector facilities using the SCC Continuous coaching, supportive supervision over long term

^a^Some participants mentioned that they did incorporate these clinical items

Of the 55 completed survey responses from implementers and non-implementers, 38 (69.1%) thought an online community of practice would be helpful in promoting and sustaining SCC use through sharing resources and facilitating connections among implementers. All interview participants thought an online community of practice would be a useful platform for implementers, policymakers, clinicians, and researchers to learn from each other. The desired functions of the community of practice included sharing lessons learned from different contexts, training in how to better advocate for resources to support SCC implementation, bridging the SCC community with other WHO initiatives and networks, and serving as a resource hub for articles and materials to improve childbirth care in multiple languages. One participant explained:

The community of practice is a good way to move forward because I have been trying to link up with people on how to push this SCC and move it forward, because right now there is no one pushing it and it’s hard to link up with anybody. I feel that people who are passionate about the SCC also don’t have that community, that network to help them get ideas on how to push it forward because right now we are just working in silos. Somebody who is passionate about it and then trying to make it work in the health facilities. Some people are succeeding and those successes need to be shared and learned in a bigger way...Having that network really brings out that you are not alone. We can share and learn together...I think one of the more successful ones are built upon this Checklist and the patient safety environment and quality of care environment. [Participant from multi-national organization]

## Discussion

Our mixed methods study presents the current landscape of SCC adaptation and implementation around the world. The vast majority of respondents indicated that they modified the SCC to reflect their local context and included both clinical and operational items. Most implementers reported that some form of initial training took place with facility staff and it included appropriate stakeholder support. Ongoing support for SCC use varied across sites based on funding and capacity constraints. Barriers to ongoing SCC implementation centered on lack of an enabling environment. Implementers desired improvements to the SCC tool and implementation strategies, such as clarifying the purpose of the SCC, developing strategies to improve adherence to practices that are difficult to change, simplifying and integrating the SCC into daily workflows, and providing ongoing support and training for SCC use. Our results show that implementation is feasible in a variety of contexts but sustaining the use of SCC over time is complicated if end users and key stakeholders are not convinced of its value. This suggests that better strategies to support long-term use of the SCC are needed to ensure its sustainability and scalability. Respondents also indicated that a community of practice would be a helpful resource for ongoing collaboration and learning. Our findings are similar to those found in the WHO Collaborative from 2012 to 2015, with an emphasis on local adaptation and ongoing support for implementation [8]. Our findings add to the literature by providing specific examples of how the SCC content, structure, and implementation have been adapted and operationalized in a variety of settings since the SCC’s public dissemination in 2015.

Promising practices around SCC adaptation include identifying the purpose of the SCC (e.g., quality improvement tool through team communication and shared accountability) as well as the desired outcomes. Adaptations to the SCC content and structure should be made in a planned, systematic way with an inclusive group of stakeholders based on the stated purpose and in accordance with local guidelines and contextual factors, such as language and respectful maternity care practices [13, 14]. Design thinking—defined as “a systematic innovation process that prioritizes deep empathy for end-user desires, needs and challenges to fully understand a problem in hopes of developing more comprehensive and effective solutions” [15]—may be particularly helpful in adapting the SCC to account for local contextual factors through ideation, prototyping, and field testing. Field testing the SCC before deploying it at scale is critical to ensuring buy-in from end-users. Promising practices around SCC implementation include defining the complementary safety bundles and other tools (i.e., partograph for labor management, complication management for hemorrhage) that need to be in place to support the SCC use, identifying additional strategies to motivate behavior change for practices that are particularly difficult to change, and addressing infrastructure constraints (lack of equipment, medications, personnel) to enhance the enabling environment before implementing the SCC.

Based on our findings, the SCC appears to be in the “early adopter” phase of the diffusion of innovation curve, which suggests that implementers—including some opinion leaders—are using the SCC because of an evaluation that the advantages outweigh the disadvantages [16]. Similar maternal safety checklists and bundles are being developed and implemented in high-income countries, such as the USA, to reduce maternal morbidity and mortality, yet uptake remains low [17]. Overcoming implementation barriers requires a foundational culture of safety that facilitates iterative improvement to boost adherence and optimize outcomes.

Our findings should be considered in the context of our study’s limitations including its relatively small sample size and potential for selection bias in how SCC implementers were identified through the Ariadne Labs network. With the sampling from Ariadne Labs’ network, we recognize that we likely did not reach all implementers of the SCC and have a skewed response from implementers proficient in English and those from non-profit organizations and academic institutions. Another limitation is our inability to reach other eligible implementers due to outdated or incorrect email addresses and the few participants from the public sector. However, we did use snowball sampling to maximize the diversity of our sample. Additionally, we reached saturation of some themes, but were unable to capture the breadth of generalizable experiences with the SCC given the small sample size.

With recent publications indicating the favorable impact of the SCC on perinatal outcomes in particular contexts [3, 4], there remains an unfinished research agenda around how to adapt and implement the SCC to optimize outcomes. An online community of practice is one platform for sharing experiences and lessons learned. Additional adaptation and implementation guidance for the SCC would be helpful for stakeholders to sustain effective implementation.

## Conclusions

Implementers of the WHO Safe Childbirth Checklist from 15 countries reported adapting the content, structure, and implementation strategies. The Checklist continues to be a tool to improve quality of care during facility-based childbirth, and implementers may benefit from additional guidance for optimal adaptation and implementation according to local context.

## Supplementary Information

**Additional file 1: Supplemental Figure 1**. Number of organizations and facilities where SCC has been implemented in the past and current use. **Supplemental Table 1**. Adaptations to SCC Content and Structure from Interviews. **Supplemental Table 2**. SCC Implementation Approaches from Interviews.

## Data Availability

De-identified survey data may be accessed by emailing the corresponding author.

## Abbreviations

WHO: World Health Organization
SCC: Safe Childbirth Checklist
