# Supplementary material for: Adaptation and implementation of the WHO Safe Childbirth Checklist around the world
Source: Implement Sci Commun. 2021 Jul 8;2:76. doi: 10.1186/s43058-021-00176-z (PMC8268383; doi:10.1186/s43058-021-00176-z)
Supplement: Supplementary file 1 — Additional file 1: Supplemental Figure 1. Number of organizations and facilities where SCC has been implemented in the past and current use. Supplemental Table 1. Adaptations to SCC Content and Structure from Interviews. Supplemental Table 2. SCC Implementation Approaches from Interviews. [file 43058_2021_176_MOESM1_ESM.docx]

**Adaptation and Implementation of the WHO Safe Childbirth Checklist Around the World**

Rose L. Molina,^1,2,3^ Anne-Caroline Benski,^4,5^ Lauren Bobanski,^1^ Danielle E. Tuller,^1^ Katherine E.A. Semrau^1,3,6^

^1^ Ariadne Labs, Harvard T.H. Chan School of Public Health / Brigham and Women’s Hospital, Boston, MA, USA

^2^ Department of Obstetrics and Gynecology, Beth Israel Deaconess Medical Center, Boston, MA, USA

^3^ Department of Medicine, Harvard Medical School, Boston, MA, USA

^4^ Department of Women, Child and Adolescent, University Hospital of Geneva, Switzerland

^5^ Takemi Program in International Health, Harvard T.H. Chan School of Public Health, Boston, MA, USA

^6^ Division of Global Health Equity, Brigham and Women’s Hospital, Boston, MA, USA

**Supplementary Materials**

Supplemental Figure 1: Number of organizations and facilities where SCC has been implemented in the past and current use


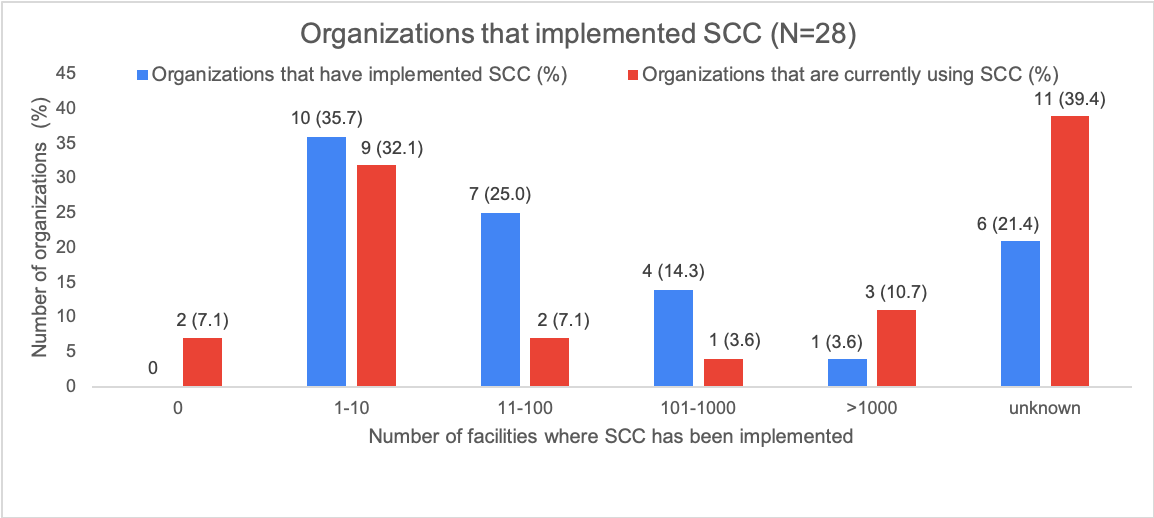


*Excluding the organization that developed a SCC mobile app.

Supplemental Table 1: Adaptations to SCC Content and Structure from Interviews

| **Adaptations to SCC Content** | **Examples** |
| --- | --- |
| Changes to clinical content | - Harmonize clinical items with national guidelines (e.g. antibiotics for prolonged ruptured membranes, removing birth companions if not allowed)  - Management of preterm births (gestational age dating, corticosteroids, antibiotics, kangaroo mother care)  - Birth companion presence  - Blood bank availability  - Patient consent  - Allergies |
| Operational items added | - Accountability (name, title, signature on each pause point)  - Additional delivery supplies and supply inventory  - Specific roles of personnel  - Government birth registration number |
| **Adaptations to SCC Structure** |  |
| Form | - Translated into local language  - Reformatted to fit onto single page  - Added WHO Safe Surgery Checklist on back of page |
| Function | - Different versions for different providers (physicians vs. midwives vs. nurses)  - Paired checklist items with clinical prompts  - SCC included in medical record  - Designated person in charge of filling out SCC |
| **Process for adapting SCC Content and Structure** | - Consultation with key stakeholders (technical advisory group, government leaders, facility leaders, multi-disciplinary clinicians), ranging from one-time meeting to continuous dialogue - Pilot study or field testing |

Supplemental Table 2: SCC Implementation Approaches from Interviews

| **Implementation Approaches*** | **Examples** |
| --- | --- |
| Initial implementation approach | Preparation  - Engaged facility leadership  - Ensured required resources were available prior to implementation |
| Initial implementation approach | Training  - One-time initial training (minimum 2 hours - maximum 5 days) or phased approach with training for different staff cadres  - Additional technical training (emergency obstetric care, simulation team training, newborn resuscitation, immunizations, breastfeeding, partograph)  - Used WHO Implementation Guide or other government program training manual  - Trained clinical managers in patient safety principles |
| Continuing implementation support | - Regular on-site mentorship/coaching  - Group text messaging thread for troubleshooting  - Refresher trainings for staff  - Regular safety or quality review meetings  - Remote mentorship/coaching (phone calls)  - No ongoing support after initial training |

*Implementation approaches listed are not mutually exclusive.
